# Successful pregnancy after in vitro fertilization-embryo transfer in a patient with cytochrome P450 oxidoreductase deficiency and a double uterus: Case report

**DOI:** 10.1097/MD.0000000000050581

**Published:** 2026-09-04

**Authors:** Shimin Wang, Xiuping Zhang, Jiayao Chen, Ningxin Zhang, Xueqing Wu

**Affiliations:** a Center of Reproductive Medicine, Children’s Hospital of Shanxi; Women Health Center of Shanxi, Shanxi Fertility Optimisation Technology Innovation Centre, Taiyuan, China; b Centre for Clinical Discipline Construction, Shanxi Medical University, Taiyuan, China.

**Keywords:** case report, cytochrome P450 oxidoreductase deficiency, in vitro fertilization, uterus didelphys

## Abstract

**Rationale:**

Cytochrome P450 oxidoreductase deficiency (PORD) is a rare autosomal recessive disorder caused by mutations in the *POR* gene, leading to impaired steroid hormone synthesis and a wide range of multisystem abnormalities. The condition is often associated with reproductive malformations and endocrine disorders, presenting clinically with symptoms such as infertility, menstrual irregularities, and adrenal dysfunction, and may be accompanied by skeletal malformations. Only a very limited number of patients have achieved successful pregnancies through assisted reproductive technology, and there is currently no standardized treatment strategy.

**Patient concerns:**

A 29-year-old woman presented with “7 years of infertility without contraception.”

**Diagnoses:**

Genetic analysis identified compound heterozygous *POR* mutations (c.1820A>G/c.1474G>A), confirming PORD. Pelvic imaging revealed uterus didelphys. Endocrine evaluation demonstrated insulin resistance and adrenal dysfunction, and the patient fulfilled the diagnostic criteria for polycystic ovary syndrome.

**Interventions:**

Following unsuccessful long-protocol ovulation induction, a modified gonadotropin-releasing hormone antagonist protocol was implemented with metformin co-treatment for insulin sensitization. Stepwise glucocorticoid therapy (oral prednisone 5 mg/day → dexamethasone 0.5 mg/day → transition to hydrocortisone 20 mg/day during pregnancy) was administered for adrenal progesterone regulation. All embryos underwent vitrification, with subsequent single frozen-thawed blastocyst transfer.

**Outcomes:**

Ovarian stimulation yielded 24 oocytes, resulting in 7 blastocysts. Successful singleton implantation was achieved. The pregnancy progressed without maternal metabolic or fetal complications, culminating in term delivery (39 weeks and 4 days) of a healthy male neonate (birth weight 3250 g, Apgar scores 9/10).

**Lessons:**

This case demonstrates that pregnancy attainment in complex PORD requires protocol individualization using gonadotropin-releasing hormone antagonists to mitigate premature luteinization, dynamic glucocorticoid titration to control adrenal steroid excess, and metabolic optimization addressing insulin resistance. The co-occurrence of *POR* mutations should be investigated in assisted reproductive technology failures with polycystic ovary syndrome-like presentations, particularly when accompanied by skeletal or genital tract anomalies.

## 1. Introduction

Cytochrome P450 oxidoreductase deficiency (PORD), a rare autosomal recessive congenital adrenal hyperplasia,^[1,2]^ results from *POR* mutations affecting its role as the electron donor for cytochrome P450 enzymes critical in steroidogenesis and drug metabolism.^[3,4]^ Since its initial identification in 2004,^[5]^ >100 pathogenic variants and 200 genetic variants have been reported,^[6]^ with both sporadic and familial cases observed despite the unclear prevalence.^[7]^ The *POR* gene (7q11.2, 32 kb) encodes an 82-kDa membrane protein (680 aa, 15 coding exons),^[3]^ essential for nicotinamide adenine dinucleotide phosphate-dependent electron transfer to steroidogenic (e.g., CYP17A1) and xenobiotic-metabolizing enzymes.^[4]^

Mutations in the *POR* gene can impair the activity of 21-hydroxylase and 17α-hydroxylase, leading to PORD. This condition exhibits significant clinical heterogeneity and is typically classified into 2 subtypes: classic and non-classic (atypical).^[2]^ Classic PORD often presents with Antley-Bixler syndrome-like skeletal malformations, glucocorticoid deficiency, and disorders of sex development.^[8]^ In contrast, atypical PORD primarily manifests with postpubertal menstrual irregularities, infertility, and ovarian cysts.^[3,9,10]^ Most patients are diagnosed in infancy or early childhood due to ambiguous genitalia or skeletal abnormalities.^[3,11]^ However, in adolescence or adulthood, symptoms such as delayed puberty, menstrual disorders, or infertility may emerge, occasionally accompanied by features of Antley-Bixler syndrome or disorders of sex development. Notably, individuals with atypical PORD are at risk of being misdiagnosed with 21-hydroxylase deficiency or polycystic ovary syndrome due to overlapping clinical features.^[12]^

A literature search using PubMed with relevant Medical Subject Headings and free-text terms revealed that, to date, no spontaneous pregnancies have been reported in women with PORD.^[3,13]^ Only 3 publications have described a total of 4 successful pregnancies achieved via in vitro fertilization (IVF) in women with atypical PORD carrying pure or compound heterozygous *POR* mutations.^[7,13,14]^ In this paper, we present the case of a Chinese woman with a compound heterozygous *POR* mutation and uterine didelphys with vaginal duplication, who successfully conceived and delivered a healthy child through IVF-embryo transfer.

## 2. Materials and methods

### 2.1. Clinical information

**Figure 1.**
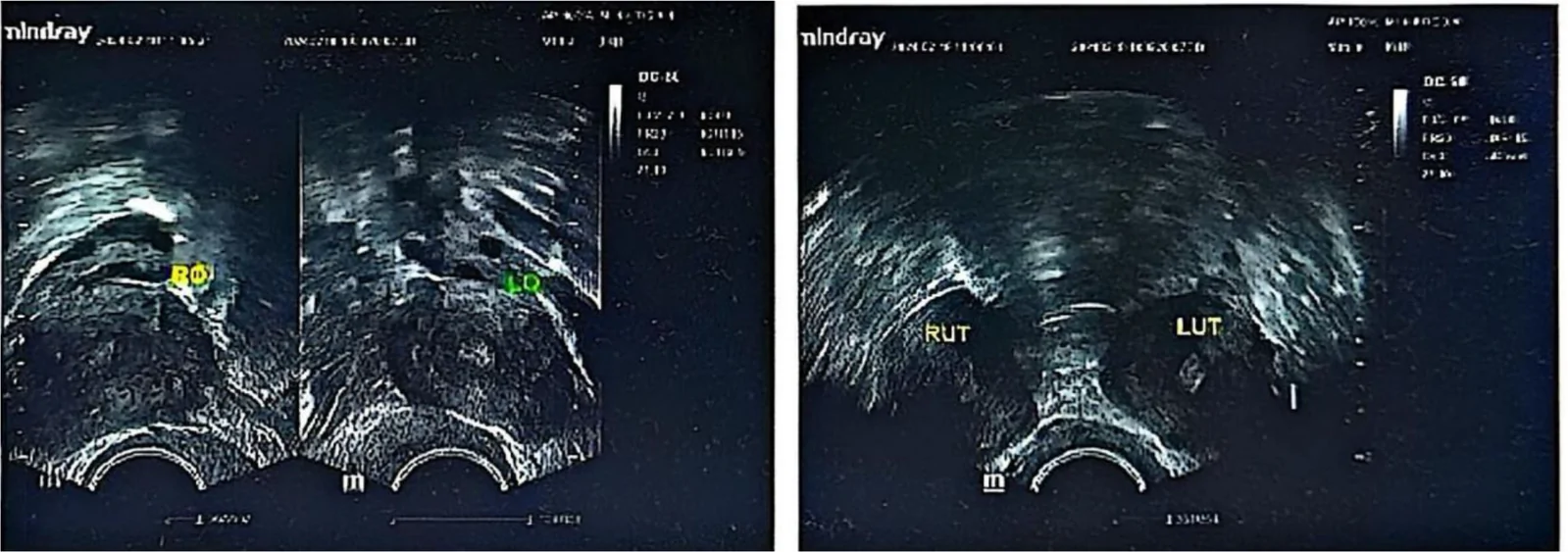
Prepregnancy ultrasound examination. LO = left ovary, LUT = left uterine tube, RO = right ovary, RUT = right uterine tube.

The patient was a 29-year-old woman who presented with a chief complaint of secondary infertility persisting for 7 years despite regular unprotected intercourse following marriage. Diagnostic evaluations identified Müllerian anomalies, specifically uterine didelphys with duplicated vaginal canals (Fig. 1). She underwent mediastinal resection in 2018 and subsequently had a vaginoplasty at our institution in 2022. Serial transvaginal ultrasonography performed between 2018 and 2022 consistently demonstrated bilateral polycystic ovarian morphology (Fig. 1). Metabolic evaluation, including insulin release testing and an oral glucose tolerance test, confirmed the presence of insulin resistance (Table 1). On physical examination, her body mass index was 27.64 kg/m^2^. She exhibited normocephalic features, with no signs of hyperandrogenism, such as acne or hirsutism, and no skeletal abnormalities were observed. Her relevant laboratory parameters are as follows (Table 2). According to the semen analysis, her husband suffered from oligospermia, with a total viability of 27%, a sperm concentration of 11.3 × 10^6^/mL, and 7.29% of the spermatozoa were morphologically normal.

**Table 1:** Basic clinical data of the patient.

Parameter	Value/description
Height (cm)	168
Weight (kg)	78
BMI (kg/m^2^)	27.64
Blood pressure (mm Hg)	110/70
Chief complaint	7 yr of infertility without contraception after marriage
Menstrual history	Menarche at age 14; cycle 30–60 d; duration 5–6 d; normal flow; no dysmenorrhea
Genital malformation	Double uterus; status post vaginal septum resection
Hyperandrogenism signs	None
Skeletal deformity	None
Ovarian cysts	None
Other conditions	Insulin resistance; obesity; polycystic ovary syndrome
RIF	Present
Gynecological ultrasound	Uterus size: 58 × 49 × 43 mm; endometrial thickness: 6.3 mm

BMI = body mass index, RIF = recurrent implantation failure.

**Table 2:** Baseline laboratory examination findings of the patient.

Parameter	Value	Normal range
C3 (g/L)	1.54	0.9–1.8
C4 (g/L)	0.23	0.1–0.4
D-dimer (µg/mL)	0.29	<0.5
Homocysteine (µmol/L)	16.8	5–15
Insulin release (µIU/mL)	Fasting (0 h): 73	2.6–24.9
	1 h postprandial: 328	Peak typically 5–10× fasting
	2 h postprandial: 217	Declines, usually <1 h value
	3 h postprandial: 84	Near/slightly above fasting
OGTT test (mmol/L)	Fasting (0 h):5	3.9–6.1
	1 h postprandial: 6.4	<10.0
	2 h postprandial: 6.7	<7.8
	3 h postprandial: 5.9	Return to fasting/slightly lower
WBC (×10^9^/L)	3.12	4.0–10.0
FSH (IU/L)	8.3	3.5–12.5
LH (IU/L)	3.4	2.4–12.6
P (ng/mL)	3.02	0.1–1.5
E2 (pg/mL)	74	12.5–166.0
17-OHP (ng/mL)	3.88	0.2–1.0

17-OHP = 17-hydroxyprogesterone, C3 = complement C3, C4 = complement C4, E2 = estradiol, FSH = follicle-stimulating hormone, LH = luteinizing hormone, OGTT = oral glucose tolerance test, P = progesterone, WBC = white blood cell.

### 2.2. Research methodology

#### 2.2.1. Genetic testing

**Figure 2.**
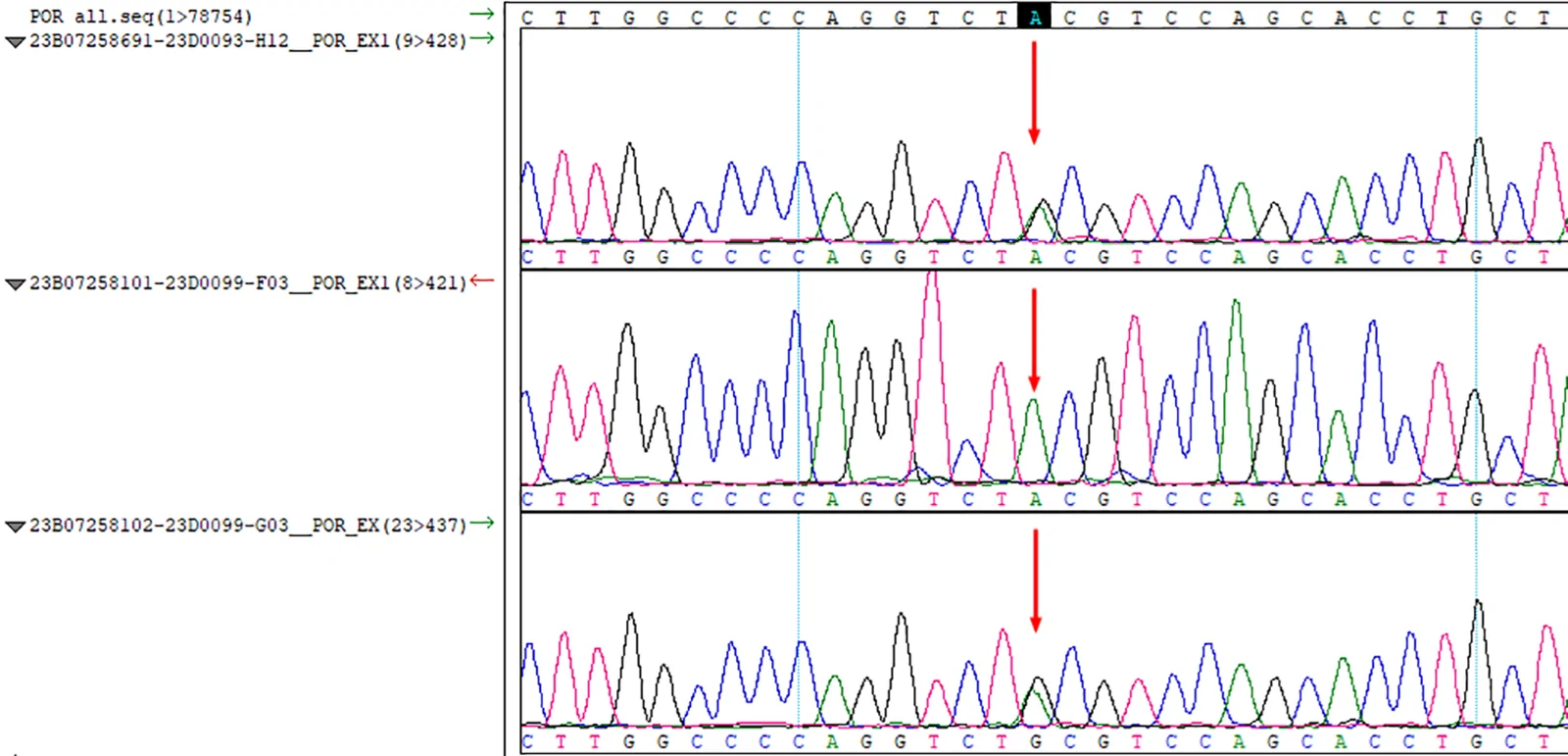
DNA sequencing chromatograms of the *POR* gene (NM_000941.2) revealing the heterozygous c.1820A>G (p.Tyr607Cys) missense variant. The arrow indicates the nucleotide substitution (A → G) at position 1820, resulting in a tyrosine-to-cysteine amino acid change at residue 607. DNA = deoxyribonucleic acid, POR = cytochrome P450 oxidoreductase.

**Figure 3.**
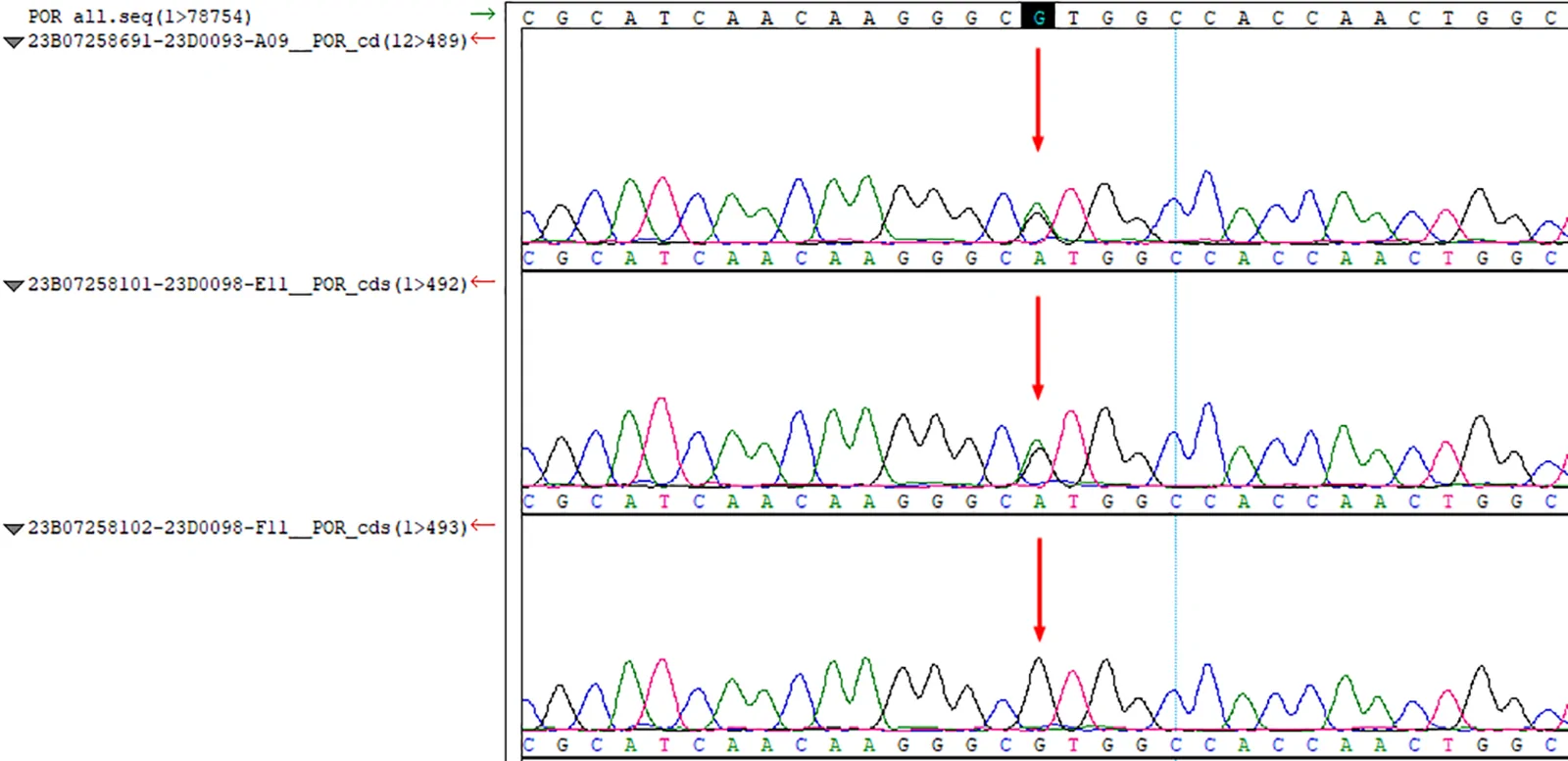
DNA sequencing chromatograms of the *POR* gene (NM_000941.2) demonstrating the heterozygous c.1474G>A (p.Val492Met) missense variant. The arrow highlights the nucleotide substitution (G → A) at position 1474, leading to a valine-to-methionine amino acid substitution at residue 492. DNA = deoxyribonucleic acid, POR = cytochrome P450 oxidoreductase.

Based on the patient’s medical history, approximately 20,000 gene exon regions and the mitochondrial genome were examined using whole-exome sequencing technology. By target region capture enrichment and high-throughput sequencing, the patient was found to carry 2 heterozygous variants in the *POR* gene: c.1820A>G (p.Tyr607Cys, suspected to be pathogenic; Fig. 2) and c.1474G>A (p.Val492Met, significance unknown; Fig. 3), both located in the exon region of chromosome 7. Sanger sequencing validation showed that the variants were inherited from the parents (paternal origin c.1820A>G and maternal origin c.1474G>A), respectively.

#### 2.2.2. Ovulation program and course of treatment

On day 1, recombinant human follicle-stimulating hormone (150 IU; Jin Sai Heng, Changchun Jin Sai Pharmaceutical Co., Ltd., China), combined with intramuscular injections of urotensin (150 IU; Lizhu Pharmaceutical Factory, Lizhu Group, China), was administered. On day 5 of the pro-ovulatory phase, gonadotropin-releasing hormone antagonism was initiated with ganirelix acetate (0.25 mg; Zhengda Tianqing Pharmaceutical Group, China), continuing through the trigger day. Progesterone levels were monitored dynamically, fluctuating between 0.69 and 2.53 ng/mL during ovulation promotion.

#### 2.2.3. Preprocessing interventions

During the preoperative conditioning phase, a split-dosing regimen was used to regulate progesterone (P) levels, with oral glucocorticoids administered as prednisone (2.5 mg at 08:00 and 1.25 mg at 16:00). After 1 month of treatment, monitoring revealed inadequate control of P levels. The prednisone dose was subsequently adjusted to 2.5 mg at both 08:00 and 16:00, combined with oral dexamethasone tablets (0.375–0.75 mg daily) to further control P levels. Following this adjustment, P levels decreased significantly to the physiological range of the early follicular phase (0.4–1.0 ng/mL). In addition, lifestyle intervention was implemented to address insulin resistance, with the patient taking metformin (0.25 g 3 times a day) with meals. Blood glucose levels were well controlled throughout the treatment.

#### 2.2.4. Transplantation and follow-up

**Figure 4.**
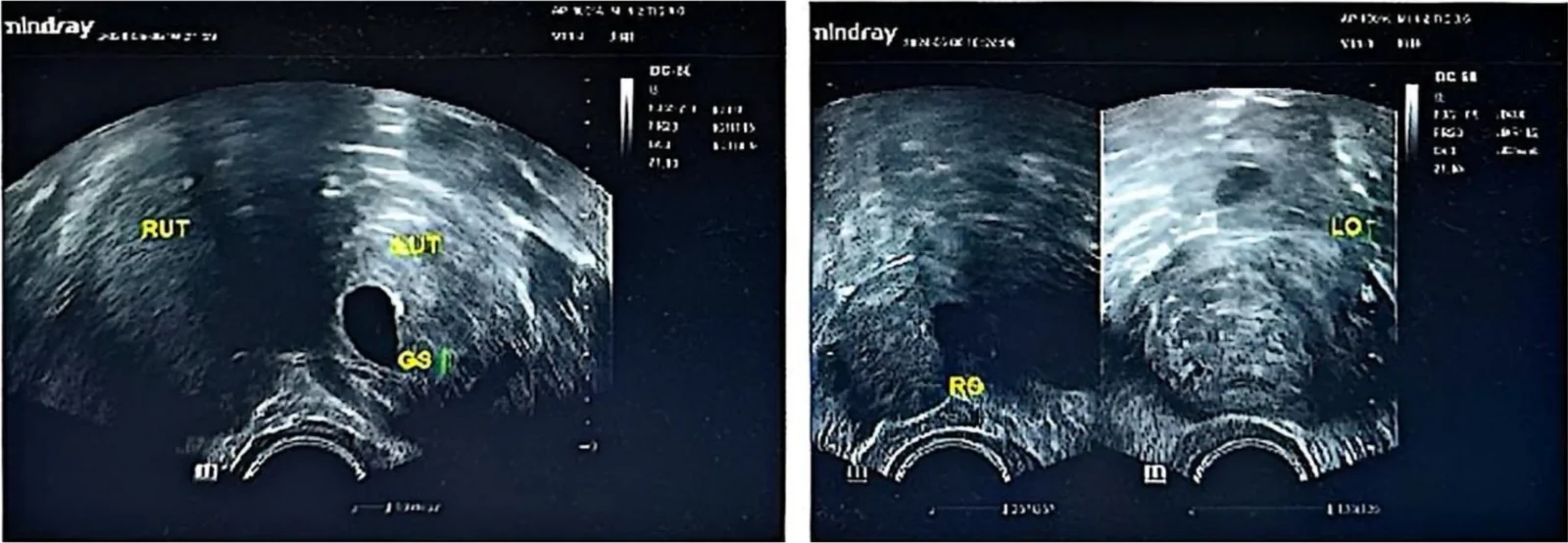
First-trimester transvaginal ultrasound confirming viable intrauterine gestation in the left cavity of a didelphic uterus.

Frozen embryos were transferred using an artificial cycle protocol. On the day of transfer, the endometrial thickness measured 9 mm, and serum progesterone was 0.62 ng/mL. A high-quality blastocyst (4AA morphology) was transferred into the left uterine cavity. Following the transfer, routine luteal support was provided until 10 weeks of gestation, which included oral hydrocortisone (10 mg in the morning, 5 mg in the evening) and prednisone (2.5 mg twice daily). At 7 days post-transfer, serum beta-human chorionic gonadotropin was 51 mIU/mL, indicating a biochemical pregnancy. By 24 days post-transfer, transvaginal ultrasonography revealed a singleton intrauterine pregnancy with a gestational sac measuring 27 × 14 mm, visible embryonic buds, and cardiac tube pulsation, confirming clinical pregnancy (Fig. 4). The ultrasonographic findings were consistent with normal early pregnancy development. Glucocorticoid therapy was gradually tapered and discontinued at 10 to 12 weeks of gestation.

#### 2.2.5. Perinatal outcome

An elective cesarean section was performed at 39^+4^ weeks of gestation. A healthy male infant was delivered, weighing 3100 g and measuring 52 cm in length, with a normal Apgar score (9/10). The neonatal physical examination showed no signs of congenital anomalies or adrenal insufficiency. 17-Hydroxyprogesterone (17-OHP) testing and genetic testing were not performed, but no evidence of adrenal insufficiency was found.

## 3. Discussion

### 3.1. Molecular basis of atypical endocrine abnormalities and diagnostic innovations

The patient displayed follicular-phase 17-OHP elevation (0.69–2.53 ng/mL) with progesterone dysregulation, indicative of non-classic congenital adrenal hyperplasia despite lacking hyperandrogenic manifestations (hirsutism/acne). Whole-exome sequencing revealed compound heterozygous *POR* mutations mechanistically linked to impaired enzymatic activity and progesterone metabolic disruption. These findings suggest a novel non-classic congenital adrenal hyperplasia subtype associated with recurrent implantation failure. We advocate adrenal axis evaluation (e.g., adrenocorticotropic hormone (ACTH)-stimulated steroid profiling).

### 3.2. Clinical misdiagnosis reflection and precision diagnosis and treatment pathway

It has been reported that many patients with PORD are diagnosed in adulthood, with diagnostic delays primarily attributed to its high phenotypic overlap with polycystic ovary syndrome. Both conditions can present with polycystic ovarian changes, menstrual disorders, and biochemical hyperandrogenism. The present case is a typical example of such a misdiagnosis. To address this challenge, we propose a 3-step differential diagnostic process: basic hormone profiling, including simultaneous testing of 17-OHP, testosterone, and the luteinizing hormone/follicle-stimulating hormone ratio during the follicular phase; standardized implementation of ACTH excitability testing; and targeted gene testing (covering key genes such as *POR* and *CYP21A2*).

### 3.3. Breakthroughs in individualized reproductive intervention strategies

In the first IVF cycle, ovulation induction with a long regimen resulted in an abnormally high progesterone level. In the second cycle, we switched to an antagonist regimen and continued metformin treatment to improve insulin sensitivity. This approach led to the retrieval of 24 eggs, with 7 high-quality blastocysts formed. Glucocorticoid regulation was carried out using a stepwise strategy: medium-acting glucocorticoid prednisone was administered prior to embryo transfer to suppress adrenal-derived progesterone, while dexamethasone was added due to inadequate control of progesterone levels. During pregnancy, the regimen was adjusted to short-acting hydrocortisone in combination with prednisone to support endometrial tolerance while minimizing the risk of fetal hypothalamic-pituitary-adrenal axis suppression. Notably, despite the *POR* mutation, oocyte quality was not significantly impacted. This finding is consistent with existing literature indicating that although PORD may lead to endometrial atrophy due to antiproliferative effects, the fertility potential of oocytes may be preserved.

### 3.4. Risk management of offspring

Although the neonatal Apgar score was normal (9/10), vigilance is necessary. PORD follows an autosomal recessive inheritance pattern, so it is recommended to perform 17-OHP testing in umbilical cord blood and conduct family *POR* gene verification. Offspring of patients with PORD may develop subclinical adrenal insufficiency. Therefore, we recommend establishing a follow-up program that should include an ACTH stimulation test during the neonatal period and evaluation of growth curves and sexual development every 2 years during childhood.

## Acknowledgments

We would like to thank the patient for participating in this study.

## Author contributions

**Funding acquisition:** Xueqing Wu.

**Investigation:** Jiayao Chen, Ningxin Zhang.

**Supervision:** Xueqing Wu.

**Writing – original draft:** Shimin Wang.

**Writing – review & editing:** Shimin Wang, Xiuping Zhang.
